# Doxycycline Usage for Prevention of Leptospirosis among Farmers and Reasons for Failure to Use Chemoprophylaxis: A Descriptive Study from Southern Sri Lanka

**DOI:** 10.1155/2019/2917154

**Published:** 2019-03-03

**Authors:** C. L. Fonseka, B. N. Vidanapathirana, C. M. de Silva, A. A. B. B. Athukorala, P. R. Goonawardena, A. P. Karunathilake, I. H. Rajapakse, A. S. Dissanayake

**Affiliations:** ^1^Department of Internal Medicine, University of Ruhuna, Sri Lanka; ^2^Teaching Hospital Karapitiya, Galle, Sri Lanka; ^3^University Medical Unit, Teaching Hospital Karapitiya, Galle, Sri Lanka; ^4^Department of Psychiatry, University of Ruhuna, Sri Lanka

## Abstract

**Background:**

Leptospirosis causes substantial morbidity and mortality in Sri Lanka. Health authorities have implemented a chemoprophylaxis programme for prevention of disease for farmers who are at a high risk of leptospirosis. Only 39% of general population is aware of chemoprophylaxis. Awareness on chemoprophylaxis and its usage among the risk population and the reasons for non-usage was uncertain. Our aim was to assess the chemoprophylaxis usage for prevention of leptospirosis among farmers and reasons for failure to use such preventive strategy.

**Methods:**

Cross-sectional descriptive study was conducted on farmers in community setting in Galle District. Multi-stage cluster sampling method was used. Out of the seventeen “Ministry of Health” (MOH) divisions within the Galle district, nine divisions were randomly selected and, subsequently, two subdivisions (“Public Health Midwife” divisions) were randomly selected from each MOH division. From each PHM division (total of 18), a cluster who does farming as the main source of income was selected. From these farmers, details on demographics, knowledge on leptospirosis and knowledge and practice on chemoprophylaxis usage were obtained through an interviewer administered open-ended questionnaire. From those who have not properly taken chemoprophylaxis, the reasons for non-usage were explored by semistructured interviews.

**Results:**

We recruited 319 (77%-males) farmers to the study. Eighteen (5.6%) have already had leptospirosis. Majority (86.8%) of farmers were aware that doxycycline can be used to prevent the disease occurrence. Only 31% knew about correct recommendations of chemoprophylaxis usage adopted by the national guidelines. Only 28.5% (91) used doxycycline prophylaxis. Out of those, only 60 farmers (65.9%) continued the prophylaxis throughout the contact and followed national recommendations. Themes responsible for non-usage were elicited such as lack of awareness of chemoprophylaxis usage, false sense of security from the disease by perceived “immunity” due to prolong exposure or due to low prevalence of disease, lack of motivation, lack of availability of medication, and fear of side effects.

**Conclusions:**

Awareness of leptospirosis is better among farmers compared to the general population. Usage of chemoprophylaxis among farmers was highly inadequate. Individual and health system related hypotheses and myths exist for non-usage of chemoprophylaxis. Thus, an urgent concerted campaign aimed at increasing awareness within the target group through education and making medicines effectively distributed is essential for better prevention of the disease.

## 1. Background

Leptospirosis is a tropical zoonotic disease which is causing substantial mortality and morbidity in Sri Lanka. In 2016, there were 4005 number of reported cases and 122 deaths and in 2018 there were 5233 notified cases [1]. Peak incidence of this disease is associated with the rice paddy-harvesting seasons, wherein an increase in the rodent population in and around the fields is observed. A significant number of patients with leptospirosis (43.5%) had been exposed in the paddy fields, indicating occupational exposure among the farmers [2]. Sri Lanka, with 28% of its growing population in the agriculture sector, has a reported annual case incidence of 5.4/100,000 population, mostly from the southern and north central regions where the disease is considered hyperendemic. Galle district falls in to southern province and has a large paddy farming population [3].

Dengue fever is another tropical infectious disease which is causing significant morbidity and mortality in Sri Lanka [4]. There has been a nation-wide media and community effort aimed at preventing the spread of Dengue fever. But, the enthusiasm from the community, media, and health planners for prevention of leptospirosis has been less evident. The reason for this is not clear. Importantly, there is convincing evidence to support that leptospirosis can be prevented in high risk population by treating them with weekly doxycycline from few studies [5–9]. Two randomized placebo control trials were conducted specifically on farmers from India and Sri Lanka using doxycycline and penicillin, respectively, where they have detected a statistically significant difference in the rates of leptospirosis and concluded that prophylaxis by either of these agents was effective in preventing disease [7, 8]. The former study has used a weekly dosage of doxycycline (200mg weekly) and the latter used a twice daily dose of penicillin (250mg bid) for a month to prevent leptospirosis. The former weekly dosing method has been used by Takafuji et al. [9] in high risk soldiers and it has considered it as a practically feasible and an effective option.

Prevention of leptospirosis by giving chemoprophylaxis (Doxycycline) is currently accepted and implemented throughout Sri Lanka [10]. The national guidelines recommend chemoprophylaxis only for well-recognized high-risk groups and doxycycline 200mg per week starting a week before an expected exposure. Thereafter, it should be continued weekly through the exposure period and continued a week after cessation of the exposure [11]. The chemoprophylaxis medication for leptospirosis is available free of charge through the hospitals, medical officer of health (MOH) office, and public health inspectors (PHI). Despite efforts on prevention including doxycycline prophylaxis, there seems to be a void in consumption of chemoprophylaxis. Also, increasing numbers of leptospirosis cases have been observed over recent years in Sri Lanka. Current data on mortality of leptospirosis gives a mean case fatality ratio of 6.85% which is the leading cause of mortality from common infectious diseases in Sri Lanka, but it may reach as high as 30-60% in complicated forms of leptospirosis [12–14].

Therefore, through the medical officer of health (MOH) officers and public health inspector's (PHI), there is an ongoing effort to put in place the practice of consumption of chemoprophylaxis in high-risk population. They educate the farmers on prophylaxis usage and distribute chemoprophylaxis to the farmers in respective areas. Agampodi et al. stated that awareness about chemoprophylaxis among the general population of Sri Lanka was 39% [15]. But, the awareness figure for the population most at risk, paddy farmers, is not clearly known. The reason for failure in translating the evidence into practice is not clear. Thus, this study is aimed at approaching the farmers to ascertain the reasons for this failure.

## 2. Methods

We carried out a cross-sectional descriptive study on paddy farmers in the community setting in Galle District. Multistage cluster sampling was used. Firstly, we randomly selected nine of the seventeen MOH (“Medical Officer of Health”) divisions of the Galle district to carry out the study. Then, from each MOH division, two “Public Health Midwife” (PHM) divisions were randomly selected, thus selecting a total of 18 PHM divisions. From each PHM division, a single cluster was selected (total of 18 clusters) with each cluster having 15-20 farming households. The authors calculated the sample size assuming 60% prevalence of doxycycline consumption with 0.05 precision. Individuals who do farming as the main source of income were selected by cluster sampling. Starting points of each cluster were selected randomly using the voters list and from there onwards each house towards the right side of the house was selected to recruit participants until the number of each cluster is reached. From each household, one adult farmer was recruited to the study. Details regarding demographics, knowledge on leptospirosis, and doxycycline usage were obtained through an interviewer-administered questionnaire by three trained researchers. The questionnaire addressed areas such as whether the subjects were aware of the risks of leptospirosis, whether they are aware that doxycycline administration may prevent the disease, whether they themselves take regular doxycycline for prevention, and if they do not, to explore the reasons for not taking the medicine.

## 3. Results

We recruited 319 farmers to the study. The majority (77%) were males. A total of 332 farmers were eligible and 319 consented to participate (consent rate was 96%). Basic demographic details are mentioned in Table 1. From the data, we observed that nearly 50 percent of farmers had reached ordinary level examination (Grade 11 in school) or above. Out of the total of 319 farmers, 18 (5.6%) has already had leptospirosis diagnosed either clinically or by confirmatory testing.

The knowledge about the main clinical features and the knowledge about organ involvement in leptospirosis among farmers are depicted in Table 3. Majority of the farmers knew that fever is a main clinical feature of leptospirosis, but other symptoms were known only by a minority. Nearly 40% farmers knew that it gives rise to kidney injury, but surprisingly 48% were unaware of any organ involvement (Table 2).

Awareness about the transmission of the disease is depicted in Table 3. We found that 10 percent of farmers were unaware of the reservoir of the illness. According to our study, most farmers knew about the disease and were aware that it was transmitted by rats through rat urine, and it enters the body through abraded skin. Only a small percentage knew that the disease could be transmitted by animals other than rats.

Knowledge and the practices with regard to the practice of using chemoprophylaxis with doxycycline are mentioned in Table 4. With regard to the knowledge about doxycycline usage as prophylaxis, 86.8% farmers knew that the drug can be used to prevent the disease occurrence and only 4% thought that boots can be used to prevent the disease. However, all farmers (100%) mentioned that wearing boots is not practical during paddy farming. Only 31% of the total number of farmers (100/319) actually knew about the correct usage recommendations. This meant that only 36% of farmers who were aware of chemoprophylaxis usage knew about the correct usage recommendations of chemoprophylaxis adopted by the national guidelines. Most of the awareness on chemoprophylaxis was given through medical personals and non-medical individuals (Table 4) and it seems that media has been able to reach only a lesser amount of high risk population.

Out of all farmers, only 91 (28.5%) used doxycycline prophylaxis as a preventive measure for leptospirosis. Out of those, only 60 farmers (65.9%) continued the prophylaxis throughout the contact in a weekly dosing regimen complying with the national recommendations [11]. Others discontinued the drug usage after a single or a few doses. There were many reasons for non-usage or discontinuation of doxycycline in the farming population. The possible themes (related to the previous instance of chemoprophylaxis usage) which came out from the semistructured discussions with the farmers were depicted in Table 5.

From the semistructured interviews conducted assessing the reasons for non-adherence or discontinuation using a semi-qualitative method, several theories for non-adherence or discontinuation were obtained (Table 5). A significant portion of farmers (27.7%) had inadequate knowledge and awareness about chemoprophylaxis. Another significant proportion of participants has not consumed it due to lack of motivation or ignorance, which could have been due to the poor understanding about the disease severity and consequences. Some had a false sense of security due to perceived “immunity” to disease due to several reasons. They thought that the overall risk of acquiring leptospirosis is low as they could be repeatedly exposed to the disease which may give “immunity” to disease or due to low prevalence of the disease in the area (Table 5).

There were several myths and beliefs we heard during the semistructured interviews which we thought were important although not being depicted in Table 5. Several thought that chemoprophylaxis is not necessary as they use pesticides in farming which kills the organism which gives rise to leptospirosis. Other than that, practical issues arose when some individuals daily went to field and conveyed that they cannot take doxycycline weekly throughout the year. On the other hand, some thought that the medication is unnecessary if they had a minimum agricultural exposure during farming as they are mostly involved in supervision of farming rather than active work. Many participants feared the side effects and cross reaction with other routine drugs. Myths about being protected in fields such as having regularly cleaned paddy fields, having no leg wounds, not getting the disease for several years meaning “immunity”, and taking medicines for other illnesses precluded chemoprophylaxis were abundant. Interestingly, two other interesting reasons emerged from the interviews where one farmer, who got complicated leptospirosis and recovered, has avoided going to the paddy field and has given up farming which was his main source of income, due to fear of the seriousness of the disease. Another farmer, who acquired leptospirosis, refused to take the medication as he developed the disease despite taking prophylaxis.

## 4. Discussion

This study was conducted to ascertain reasons for failure to translate evidence into practice in the use of doxycycline for prophylaxis against leptospirosis, in a high-risk population, the rural farming community in Sri Lanka. Though convincing evidence on effectiveness of prophylaxis on risk population and a national policy mentioning the chemoprophylaxis usage in this population exist, it has not worked well as it should.

Our findings indicate the following: though the level of awareness and access to prophylactic medicine is at a satisfactory level, the true barriers to translation of evidence into practice stem from myths, misconceptions, and a general lack of insight about the benefits. In more than 75% of instances, the reason for non-adherence with doxycycline prophylaxis has been lack of awareness that these medicines could prevent the disease, false beliefs such as perceived “immunity” from previous episodes of exposure to leptospirosis or short-term exposure not causing the disease or if one has not got in the years gone-by that one is unlikely to get it now, perceived low risk due to assumed low prevalence, and the medicines carrying harmful side effects.

Previous research regarding knowledge of doxycycline prophylaxis use in Sri Lanka comes from data gathered from the general population. It is unlikely that those who do not perceive themselves to be directly affected by a health condition would take more than a cursory interest in health recommendations. Thus, an awareness level of 39% in general population was supplanted as expected [12], by a higher awareness level of 86% in our study on the farming population. Despite this high awareness, the actual usage was one-third, at less than 30%. This indicates that there is indeed problem that needs to be rectified urgently.

Raising general levels of health literacy in a community are a long drawn-out process which will involve educational, economic, and social condition improvement in the society. The knowledge generated by this study calls for a focused action plan to engage the farming community, specifically. Awareness creation about doxycycline prophylaxis and making it accessible to the community have reached satisfactory levels but much remains to be done in improving awareness and commitment of the farming community to take the medicine according to the national policy. A concerted campaign aimed at dispelling myths and correcting misbeliefs which is carried out by public health officers at village farmer committee level may address the problems highlighted in the study. If the adherence level can be improved to match the awareness level, it is likely that there will be a substantial improvement in disease burden levels.

The study was based in the susceptible community, speaking to farmers who were at risk of the disease. The sample was restricted to the southern province and farming occurs in many other high-risk areas and it is possible that the reason for failure to translate national health policy into practice may be different in other regions and different methods of addressing the problem may be needed.

Further research is needed in several aspects. Other farming areas of the country which may have different ethnic variations and farming practices with vulnerable populations may need to be investigated to assess adherence and if the adherence levels are low, to ascertain the reasons as to whether they are the same as identified here or whether different behavioural dynamics are in operation. Research is needed to identify methods for further improvement of awareness in the farming community and methods to correct misconceptions. Whether meetings with local farming village committees or use of information leaflets or use of mass media or whether the use of a combination of these methods is better could be identified with further research.

What is clearly evident is that there is an urgent need to have an action plan to address the areas of concern identified in the study, namely, the low levels of adherence and the myths and misconceptions acting as barriers to improving adherence.

## 5. Conclusions

Although awareness of leptospirosis is better among farmers compared with the general population, the usage of chemoprophylaxis remained at an inadequately low level. Several individual and health system related themes exist which has led to failure of consumption of chemoprophylaxis. Thus, this need for an urgent concerted campaign aimed at increasing awareness within the target group through education, changing the perception of illness by explaining the myths, and making medicines freely accessible whilst minimizing the impact of side effects is essential for better prevention of the disease.

## Figures and Tables

**Table 1:** Sociodemographic characteristics of the study sample.

	number	%
*Gender*		

Male	246	77.1 (72.2-81.3)

Female	73	22.9 (18.6-27.8)

*Educational level*		

Primary	57	17.9 (14.05-22.4)

Secondary	99	31.0 (26.2-36.3)

Ordinary level (Grade 11)	106	33.2 (28.2-38.5)

Advanced level (Above Grade 11)	50	15.7 (12.1-20.07)

Graduate	7	2.2 (1.07-4.4)

*Type of farming*		

Paddy field	295	92.5 (89.05-94.8)

Chena cultivation (Shifting agriculture)	24	7.5 (5.1-10.9)

**Table 2:** Knowledge about the main clinical features and organ involvement in leptospirosis among farmers: frequency of how much people knew about each parameter.

	Number	%
*Awareness of Symptoms/signs*		

Fever	248	77.7 (72.8-81.9)

Headache	67	21.0 (16.8-25.8)

Muscle pain	83	26.0 (21.5-31.1)

Low urine output	43	13.5 (10.1-17.6)

Yellow discoloration of eyes	25	7.8 (5.36-11.3)

Red bleeding areas in the eye	82	25.7 (21.2-30.7)

Don't know	33	10.3 (7.46-14.17)

*Awareness about organ/system involvement*		

Kidney	126	39.5 (34.2-44.9)

Liver	39	12.2 (9.07-16.2)

Blood related	48	15.0 (11.5-19.3)

Lungs	31	9.7 (6.93-13.4)

Nervous system related	39	12.2 (9.07-16.2)

Don't know about any organ/system involvement	153	48.1 (42.5-53.4)

**Table 3:** Knowledge about the transmission of leptospirosis among farmers.

	Number of farmers who had the knowledge	%
Have heard about the disease	319	100

*Knowledge about the Reservoir *		

Rat	284	89.0 (85.1-92.0)

Cattle	11	3.4 (1.94-6.07)

Other	2	0.6 (0.1-2.2)

Don't know	33	10.3 (7.46-14.1)

*Mode of exposure to disease*		

Contact with contaminated mud or water by rat urine	216	67.7 (62.4-72.6)

direct contact with rat urine	83	26.0 (21.5-31.1)

Working in paddy field	4	1.3 (0.4-3.1)

Don't know	12	3.8 (2.1-6.4)

*Entry to disease to the body*		

Through wounds	258	80.9 (76.2-84.8)

Mucous membrane	35	11.0 (8-14.8)

Undamaged skin	29	9.1 (6.4-12.7)

Don't know	48	15.0 (11.3-19.3)

*High mortality from disease*		

Yes	288	90.3 (86.5-93.0)

No	25	7.8 (5.3-11.3)

Don't know	5	1.6 (0.6-3.6)

**Table 4:** Knowledge and awareness of farmers on modes of prevention of the disease, practice of using chemoprophylaxis with doxycycline.

	Number	%
*Prevention of disease*		

Using chemoprophylaxis medication	277	86.8 (82.6-90.1)

Knew correct chemoprophylaxis usage recommendations of national guidelines	100	31.3 (26.5-36.6)

Using boots	13	4.1 (2.4-6.8)

Kill rats	9	2.8 (1.4-5.2)

*Source of information about chemoprophylaxis (multiple choices)*		

Newspaper	52	

Radio	27	

Television	74	

Medical personal	137	

Non-medical / hear say	118	

Don't know	22	

*Source of obtaining chemoprophylaxis (multiple choices)*		

Public health inspector	77	

Medical officer	43	

Pharmacy	184	

“Farmers' welfare society”	40	

Don't know	58	

**Table 5:** Reasons for non-adherence or discontinuation of doxycycline prophylaxis.

Reasons for non-adherence	Number	%
Lack of awareness or knowledge on chemoprophylaxis usage	72	27.7

False sense of security by perceived “immunity” to disease	60	23.1

Low disease prevalence in the community	41	15.8

Lack of motivation	30	11.5

Lack of availability of chemoprophylaxis (due to problems of distribution of drug)	21	8.1

Fear of side effects and drug interactions	14	5.4

Preference to alternative medicine	13	5.0

Other	8	3.0

Total	259	99.6

## Data Availability

The datasets used and/or analyzed during the current study are available from the corresponding author on reasonable request.

## Abbreviations

MOH: Medical officer of health
PHI: Public health inspector
PHM: Public Health Midwife.
